# Brain microglia activation and peripheral adaptive immunity in Parkinson’s disease: a multimodal PET study

**DOI:** 10.1186/s12974-022-02574-z

**Published:** 2022-08-29

**Authors:** Shu-Ying Liu, Hong-Wen Qiao, Tian-Bin Song, Xiu-Lin Liu, Yun-Xia Yao, Chun-Song Zhao, Olivier Barret, Sheng-Li Xu, Yan-Ning Cai, Gilles D. Tamagnan, Vesna Sossi, Jie Lu, Piu Chan

**Affiliations:** 1 Department of Neurology, Xuanwu Hospital, Capital Medical University, Changchun Street 45, Beijing, 100053 China; 2 Department of Radiology and Nuclear Medicine, Xuanwu Hospital, Capital Medical University, Beijing, China; 3 Department of Neurobiology, Xuanwu Hospital, Capital Medical University, Beijing, China; 4 Chinese Institute for Brain Research (CIBR), Beijing, China; 5 Laboratoire des Maladies Neurodégénératives, Université Paris-Saclay, CEA, CNRS, MIRCen, Fontenay-Aux-Roses, France; 6 National Clinical Research Center for Geriatric Diseases, Beijing, China; 7 Mental Health PET Radioligand Development (MHPRD) Program, Yale University, New Haven, USA; 8 Department of Physics and Astronomy, University of British Columbia, Vancouver, BC Canada

**Keywords:** Parkinson’s disease, Microglia, Cytokine, Th cell, Lymphocyte, Positron emission tomograph

## Abstract

**Background:**

Abnormal activation of immune system is an important pathogenesis of Parkinson’s disease, but the relationship between peripheral inflammation, central microglia activation and dopaminergic degeneration remains unclear.

**Objectives:**

To evaluate the brain regional microglia activation and its relationship with clinical severity, dopaminergic presynaptic function, and peripheral inflammatory biomarkers related to adaptive immunity.

**Methods:**

In this case–control study, we recruited 23 healthy participants and 24 participants with early-stage Parkinson’s disease. ^18^F-PBR06 PET/MR for microglia activation, ^18^F-FP-DTBZ for dopaminergic denervation, total account of T cells and subpopulations of T helper (Th1/Th2/Th17) cells, and the levels of serum inflammatory cytokines were assessed. Sanger sequencing was used to exclude the mix-affinity binders of ^18^F-PBR06-PET.

**Results:**

Compared to healthy controls, patients with Parkinson’s disease had an increased ^18^F-PBR06-PET standardized uptake value ratio (SUVR) in the putamen, particularly in the ipsilateral side of the motor onset. ^18^F-PBR06-PET SUVR was positively associated with ^18^F-FP-DTBZ-PET SUVR in the brainstem and not associated with disease severity measured by Hoehn and Yahr stage, MDS-UPDRS III scores. Patients with Parkinson’s disease had elevated frequencies of Th1 cells and serum levels of IL10 and IL17A as compared to healthy controls. No significant association between peripheral inflammation markers and microglia activation in the brain of PD was observed.

**Conclusion:**

Parkinson’s disease is associated with early putaminal microglial activation and peripheral phenotypic Th1 bias. Peripheral adaptive immunity might be involved in microglia activation in the process of neurodegeneration in PD indirectly, which may be a potential biomarker for the early detection and the target for immunomodulating therapy.

**Supplementary Information:**

The online version contains supplementary material available at 10.1186/s12974-022-02574-z.

## Introduction

The pathophysiology of Parkinson’s disease (PD) results from a complex interplay among α-synuclein aggregation, neuroinflammation, and dysfunction of mitochondria, lysosomes and synaptic transport [1]. The presence of activated microglial cells was observed in the substantia nigra and putamen in postmortem studies of PD, supporting the role of central neuroinflammation [2, 3]. In addition, aberration in peripheral inflammatory cytokine levels and immune cell counts were demonstrated in blood and cerebrospinal fluid from PD patients [4, 5], suggesting the involvement of peripheral innate and adaptive immunity in the degenerative process of PD. However, associations between peripheral inflammation, central microglia activation and dopaminergic degeneration have not been clearly described in PD.

Recent studies have showed the infiltration of T lymphocytes (CD4+ and CD8+) but not B lymphocytes in the affected brain regions in PD [6], and T cells from patients with PD could recognize alpha-synuclein peptides [7]. Adoptive transfer of both T helper 1 (Th1) and Th17 cells significantly potentiated the degree of neurodegeneration in mice model, and the IL-17-producing Th17 cells displayed the highest potency [8]. Moreover, Th17 cells isolated from PD patients could provoke midbrain neurons derived from induced pluripotent stem cells to undergo cell death [9], indicating a key role of T lymphocytes in PD pathogenesis.

The 18-kDa translocator protein (TSPO), is known to be expressed in glia cells including microglia and astrocytes, and can be quantified using positron emission tomography (PET) and thus, can be considered a putative biomarker of microglia activation in vivo [10, 11]. Several studies have observed an increase of TSPO binding in the midbrain, striatum or cortex of PD mainly using the first-generation TSPO tracer ^11^C-(R)-PK11195 [12–16] or the second-generation tracers ^11^C-DPA713/^18^F-DPA714 [17, 18], while some other studies showed a lack of binding differences between PD and healthy controls using the second-generation TSPO tracers ^18^F-FEPPA or ^11^C-PBR28 [19–21]. Studies also evaluated the associations between microglia activation and dopaminergic denervation measured by either tracers of dopaminergic transporter or Fdopa, which reported inconsistent results [12, 15, 18, 21].

Taking advantage of lower impact of the *rs6971* polymorphism of TSPO gene in the Chinese population, the current study has evaluated (i) the central microglia activation in PD using the second-generation TSPO radioligand ^18^F-PBR06; (ii) the relationship between central microglia activation, disease severity and dopaminergic denervation; (iii) the associations between the central microglia activation and peripheral inflammatory.

## Materials and methods

### Participants and clinical assessments

Participants were recruited between September 2019 to January 2021 in Xuanwu Hospital, Capital Medical University. PD participants were diagnosed by movement disorder specialists according to the MDS clinical diagnostic criteria for PD [22]. Patients with dementia, neuropsychiatric disease or receiving anti-inflammation drugs, i.e., aspirin, were excluded. The genotyping of *rs6971* polymorphism was carried out with sanger sequencing [23]. Participants carrying the TSPO *rs6971* A/G were mixed-affinity binders, while those with G/G, were defined as high-affinity binders. According to the 1000 Genomes Project Phase 3, the proportion of high-affinity binder is around 95% for the Chinese population; thus, only high-affinity binders were included in the finial statistical analysis. The study was approved by the Clinical Research Ethics Board of Xuanwu Hospital and all participants provided written informed consent.

Neurological assessments including Hoehn and Yahr stage, MDS-Unified Parkinson’s Disease Rating Scale (MDS-UPDRS), Mini-Mental State Examination (MMSE), Montreal Cognitive Assessment (MoCA), Geriatric Depression Scale (GDS), REM Sleep Behavior Disorder Questionnaire-Hong Kong (RBDQ-HK) were performed, the Hoehn and Yahr stage and MDS-UPDRS were preformed in OFF state in PD patients (off medication for at least 12 h). Venous blood samples were obtained from all participants on the same day before ^18^F-PBR06 PET scan.

### Measurement of cytokines in the plasma

Venous blood was centrifuged at 2500×*g* for 10 min at 4 °C within 30 min of blood collection. The extracted plasma sample was aliquoted and stored at − 80 °C until analysis. V-PLEX Custom Human Biomarkers Kits (K151A9H) were used to measure 11 cytokines simultaneously, according to the manufacturer’s instructions (Meso Scale Discovery, Rockville, MD, USA). Discovery Workbench software (version 4.0.12; Meso Scale Diagnostics, LLC) was used to establish calibration curves and determine cytokine levels as reported previously [24].

### Flow cytometric analysis of CD4^+^ T helper cells in blood

Peripheral blood mononuclear cells were separated using Ficoll density gradient centrifugation within 6 h, and stored in cell cryopreservation box at 4 °C for 30 min and − 80 °C overnight, then transferred to liquid nitrogen for storage. Peripheral blood mononuclear cells were resuscitated in a 37 °C water bath and washed with PBS, Leukocyte Activation Cocktail was added for stimulation and FVS510 to identify dead cells. All the samples were incubated with a cocktail of anti-human CD3, CD4, CD8 antibodies at room temperature for 20 min in the dark, and washed twice with PBS. After washing, BD cytofix/cytoperm fixation/permeabilization solution kit was used to break the membrane and fix the cells for 20 min according to the instructions. IL-4-APC, IFN-γ-FITC and IL-17A-PE were used for intracellular staining, incubated in the dark at room temperature for 20 min. The cells were resuspended in 1% paraformaldehyde. After staining, all samples were collected on the BD Canto II flow cytometer, and analyzed with Flowjo 7.6.1 software. Th cells were delineated by the membranous CD3 and CD4 expression. Th cell subpopulations were separated based on the staining results of IFN-γ, IL-4, and IL-17A: Th1 cells (CD4^+^ IFN-γ^+^), Th2 cells (CD4^+^ IL-4^+^), and Th17 cells (CD4^+^ IL-17A^+^).

### Production of radiopharmaceuticals

Two PET imaging tracers were used in the study: ^18^F-FP-DTBZ for vesicular monoamine transporter 2 (dopaminergic denervation) and ^18^F-PBR06 for TSPO (microglia activation). Both were prepared from the corresponding tosyl precursors through a one-step nucleophilic substitution fluorination reaction with no-carrier-added ^18^F, followed by purification with semi-preparative high-performance liquid chromatography [25]. The preparation processes were performed on Neptis perform synthesizer with disposable cassettes. Each tracer was formulated in a mixture of ethanol (< 12%) and 0.5% sodium ascorbate saline solution before final sterilization by 0.22 μm filtration (Millex GV filter, Millipore).

### PET/MR scanning

PET/MR scans were performed on a hybrid 3.0-T PET/MR scanner (uPMR790, UIH, Shanghai, China) with a voxel size of 1.82 × 1.82 × 2.78 mm [26]. A single frame was acquired between 60 and 90 min after an intravenous bolus injection of 370 MBq ^18^F-PBR06. Similarly, a single frame was acquired between 90 and 105 min after an intravenous bolus injection of 222 MBq ^18^F-FP-DTBZ. The ^18^F-FP-DTBZ PET/MR scans were performed in OFF state in PD patients. The ^18^F-FP-DTBZ and ^18^F-PBR06 scans were performed within a week for all participants. The subject’s head was stabilized with foam pads to reduce motion. During PET data acquisition, two anatomical brain MRI scans were performed. T2-weighted imaging was used for clinical evaluation and exclusion of pathology. 3D T1-weighted imaging was used for co-registration with the PET images and spatial normalization [27].

### Image analysis

The PET/MR frames were spatially segmented, co-registered and normalized for each subject using the PNEURO utilities suite. A standardized set of regions of interest was automatically placed on bilateral caudate, putamen, substantia nigra and brainstem using Hammer’s atlas. The putamen was further divided into the anterior dorsal part, anterior ventral part, the posterior dorsal part, and the posterior ventral part. Regional standardized uptake value ratio (SUVR) for ^18^F-PBR06-PET was calculated for each participant based on normalization of the individual region’s SUV to the global brain SUV [28, 29]. To assess the appropriateness of using global SUV as a reference for normalization, global SUV was compared between the PD and HC groups and showed no significant difference (1.93 ± 0.54 vs. 2.00 ± 0.77, *p* = 0.751). Regional SUVR for ^18^F-FP-DTBZ-PET were calculated for each participant using the occipital cortex as reference. Due to the asymmetric feature of neurodegeneration in PD, regional analysis was applied in which ipsilateral and contralateral sides were referred to the hemisphere associated with first onset of clinical symptoms.

### Statistical analyses

Independent two-tailed *T*-test was used for comparisons of age, MDS-UPDRS III scores, MMSE, and MoCA between PD and control, and a binomial variable with Fisher’s exact test was used for gender. GDS and RBDQ-HK were compared using Mann–Whitney test. The SUVR of ^18^F-PBR06-PET was analyzed using independent two-tailed *t* test and covariance was adjusted for age and gender. We compared the level or percentages of the peripheral markers using *T*-test or Mann–Whitney test. Linear regressions were applied for correlation between ^18^F-PBR06-PET SUVR and disease duration, MDS-UPDRS and ^18^F-FP-DTBZ-PET SUVR. Pearson’s or Spearman correlation was applied between ^18^F-PBR06-PET SUVR and peripheral inflammatory makers depending on the distributions. *P* values less than 0.05 were set as significantly different. The statistical analysis was done with SPSS for Windows, version 19.0 (SPSS, Chicago, IL, USA). Multiple comparisons were corrected by controlling the false discovery rate using the Benjamini–Hochberg procedure.

## Results

### Clinical characteristics

Among 24 PD participants and 23 healthy controls recruited, only 2 PD and 1 healthy control were mix-affinity binders for the *rs6971* polymorphism of TSPO gene and were excluded for analysis. The demographic and clinical characteristics are shown in Table 1. No differences in age and gender were noted between PD patients and controls, and no adverse events occurred during the PET scans. The PD patients were in the early disease stage with an average disease duration 2.5 years (30.18 ± 16.59 months) after any self-reported motor symptoms and a Hoehn and Yahr stage of two (1.82 ± 0.57). No between-group differences were found in GDS, MMSE, MoCA or RBDQ-HK (Table 1).

**Table 1:** Demographic and clinical features

	Healthy control	Parkinson’s disease	*P*	Healthy control	Parkinson’s disease
	High-affinity binders			Mix-affinity binders	
No.	22	22	–	1	2
Age	58.91 ± 9.78	59.27 ± 11.96	0.913	59	60, 66
Gender (M/F)	10/12	15/7	0.128	0/1	1/1
MDS-UPDRS III scores	0.41 ± 1.33	21.23 ± 10.14	< 0.0001	0	11, 5
GDS	6.09 ± 6.29	6.27 ± 5.44	0.680^a^	2	7, 16
MMSE	28.45 ± 1.79	26.95 ± 6.35	0.236	30	28, 30
RBDQ-HK	6.95 ± 8.28	12.73 ± 14.71	0.163^a^	5	4, 9
LEDD	–	508.33 ± 239.7	–	–	0, 0
Dose (^18^F-FP-DTBZ)	283.82 ± 60.34	298.00 ± 67.01	0.469	346	267, 253
Dose (^18^F-PRB06)	348.14 ± 19.61	353.35 ± 21.54	0.430	360	373, 332

*GDS* Geriatric Depression Scale, *MDS-UPDRS III* MDS-Unified Parkinson’s Disease Rating Scale motor scores, *MMSE* Mini-Mental State Examination, *MoCA* Montreal Cognitive Assessment, *P* *p*-value, *RBDQ-HK* REM Sleep Behavior Disorder Questionnaire-Hong Kong, *SUVR* standardized uptake value ratio

^a^Calculated with Mann–Whitney test

### Increase of microglia activation in the basal ganglia of PD

**Fig. 1:**
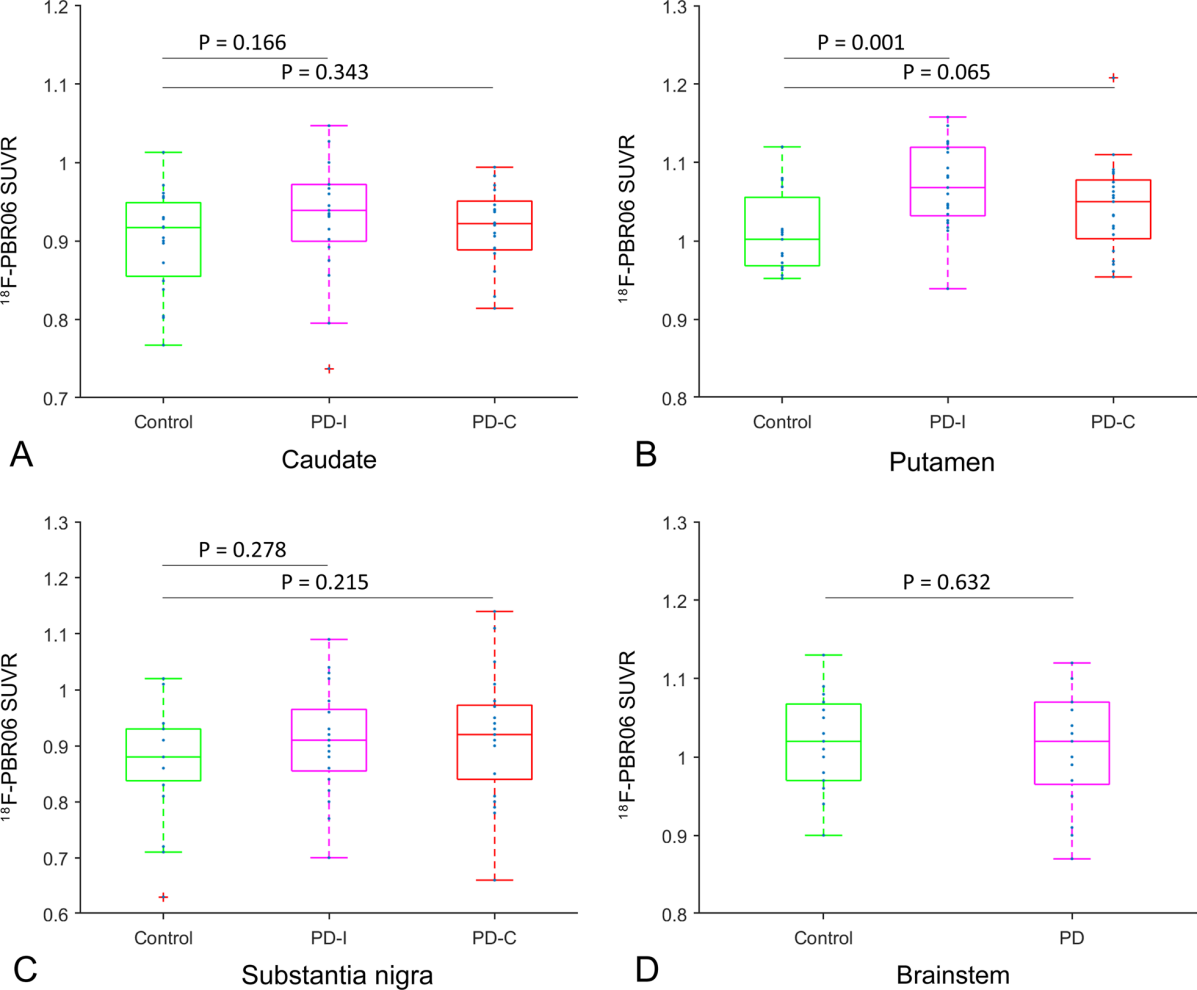
Neuroinflammation in striatum subregions, substantia nigra and brainstem. The SUVR of ^18^F-PBR06 between patients with Parkinson’s disease and healthy controls in the **A** caudate, **B** putamen, **C** substantial nigra, **D** brainstem. *C*Contralateral side to the onset side of motor symptoms. *I* Ipsilateral side to the onset side of motor symptoms. *PD* Parkinson’s disease, *SUVR* standardized uptake value ratio

Comparing to healthy controls, the ^18^F-PBR06-PET SUVR was higher in the putamen, but statistically only on the ipsilateral to the side first observed motor symptoms of PD (Fig. 1). The increase of ^18^F-PBR06 SUVR remained significant after controlling for age and gender and survived the correction for multiple comparisons (Additional file 1: Table S1). No differences were observed in the caudate nucleus or substantia nigra (Fig. 1). Further post hoc analyses on the putaminal subregions showed significant increase of the ^18^F-PBR06-PET SUVR in all subregions of putamen on the ipsilateral side but only in the posterior ventral putamen on the contralateral side (Additional file 1: Table S2).

### Association between brain microglia activation, disease severity and dopaminergic presynaptic disruption

No significant correlations were found between ^18^F-PBR06-PET SUVR, disease severity as measured by Hoehn and Yahr stage and MDS-UPDRS III scores in all subregions of striatum and substantia nigra (Table 2). However, the ^18^F-PBR06-PET SUVR of brainstem was negatively associated with MDS-UPDRS III score (*R* = − 0.452, *P* = 0.040), and positively associated with dopaminergic function measured by ^18^F-FP-DTBZ SUVR.

**Table 2:** Linear regressions between microglia activation, disease duration, UPDRS III, and dopaminergic disruption in PD

VOI	Side		Duration	Hoehn and Yahr stages	MDS-UPDRS III scores	^18^F-FP-DTBZ SUVR
Caudate	Ipsilateral	*R*	− 0.163	0.003	− 0.288	− 0.042
		*P*	0.480	0.990	0.206	0.862
Putamen		*R*	− 0.184	0.398	0.412	0.204
		*P*	0.424	0.074	0.063	0.389
Substantia nigra		*R*	0.007	− 0.206	− 0.403	0.148
		*P*	0.975	0.370	0.070	0.533
Caudate	Contralateral	*R*	− 0.019	− 0.023	− 0.366	− 0.141
		*P*	0.935	0.920	0.103	0.553
Putamen		*R*	− 0.138	0.421	0.349	0.124
		*P*	0.551	0.058	0.121	0.603
Substantia nigra		*R*	− 0.194	0.017	− 0.400	0.187
		*P*	0.399	0.940	0.072	0.430
Brainstem		*R*	0.269	− 0.140	− 0.452	0.688
		*P*	0.238	0.544	0.040*	0.001*

*R* correlation coefficient, *P* *p*-value, *MDS-UPDRS III* MDS-Unified Parkinson’s Disease Rating Scale motor scores, *SUVR* standardized uptake value ratio

*P < 0.05

### Peripheral inflammation in PD

The count of total T cells, and frequencies of Th2 or Th17 cells among T cells in blood did not differ between PD patients and healthy participants, while the frequencies of Th1 cells among total T cells was increased in PD comparing to controls (12.85 ± 3.19% vs. 9.81 ± 2.42%, *P* = 0.008), Th2 cells slightly decreased and Th17 cells increased, respectively (Table 3). For the cytokines, levels of cytokines IL17A (0.577 ± 0.352 vs. 0.395 ± 0.204, *P* = 0.045) and IL10 (0.202 ± 0.195 vs. 0.083 ± 0.052, *P* = 0.002) were significantly higher in patients with PD than that of healthy controls, and the increase of IL10 level survived the correction for multiple comparisons.

**Table 3:** Peripheral inflammation in the blood of PD patients and healthy controls

	Control *n* = 22	PD *n* = 22	*P*
Total T cells/lymphocytes	73.24% ± 8.78%	72.95% ± 7.47%	0.976
Th1/total T cells	9.81% ± 2.42%	12.85% ± 3.19%	0.008*
Th2/total T cells	3.25% ± 2.51%	3.15% ± 1.46%	0.842
Th17/total T cells	1.65% ± 0.79%	2.10% ± 1.53%	0.743
IFNγ	6.30 ± 2.63	5.62 ± 2.35	0.375
TNFα	1.88 ± 0.38	1.92 ± 0.42	0.707
IL6	0.746 ± 0.462	0.659 ± 0.432	0.343^a^
IL8	7.02 ± 2.86	7.89 ± 2.82	0.320
IL10	0.083 ± 0.052	0.202 ± 0.195	0.002*^a^
IL12p70	0.078 ± 0.117	0.070 ± 0.049	0.762
IL22	0.605 ± 0.652	0.440 ± 0.354	0.921^a^
IL17A	0.395 ± 0.204	0.577 ± 0.352	0.045*

*P* *p*-value, *PD* Parkinson’s disease, *Th* T helper cells

*P < 0.05

^a^Calculated with Mann–Whitney test

### Associations between peripheral inflammation and brain microglia activation in PD

In general, there was no significant association between levels of microglia activation in the brain and peripheral inflammation markers. Associations were noted within the ^18^F-PBR06 SUVR of regions in the central nervous system, and within the levels of peripheral markers, but not in-between them (Additional file 1: Fig. S1).

## Discussion

Using ^18^F-PBR06-PET SUVR, a marker of TSPO mainly representing brain microglia activation, the current study found increased regional microglia activation in the putamen and relatively increased blood subpopulation of Th1 lymphocytes and levels of IL10 and IL17A cytokines in patients with early PD. The increase of activated microglia was more prominent in the ipsilateral side of the motor onset, and not associated with disease severity measured by Hoehn and Yahr stage, MDS-UPDRS III scores, and dopaminergic terminal density. No significant association between peripheral inflammation markers and microglia activation in the brain of PD was observed. Our data support the notion that adaptive immunity is involved in the early phase of neurodegenerative processes.

TSPO-PET has been deemed a surrogate marker of neuroinflammation levels since the level of TSPO significantly increase in microglia upon activation. Two generations of TSPO tracers were developed to date. The first-generation TSPO tracer ^11^C-PK111195 was the most widely used so far, but suffered from non-specific bindings and low brain uptake. Several second-generation tracers (^11^C-PBR28, ^18^F-PBR06, ^11^C-DPA713, ^18^F-DPA714, ^18^F-FEPPA) was developed later with better binding potential to TSPO, but the uptake of the tracers was soon found to be significantly influenced by the *rs6971* polymorphism of TSPO [23]. Interestingly, studies with the application of the first-generation TSPO tracer ^11^C-PK111195 showed an increase of microglia activation in the brain of PD patients uniformly [12–16], while recent findings of studies with the second-generation TSPO tracers were less consistent [17–21]. One possible explanation is that approximately 40–45% of the Caucasian population are high-affinity binders and 40–45% are median-affinity binders, which significantly dilute the power of statics analysis. Taking advantage that the difference on *rs6971* polymorphism is rare in Asian populations, we have observed an increase of microglia activation in the putamen of PD patients in a genetically homogeneous group of participants for TSPO polymorphism in this study. Our results further support the involvement of microglia activation in early PD using the second-generation TSPO tracer.

Microglia activation was found selectively increased in the putamen of early-stage PD patients, particularly in the potentially mild lesion side ipsilateral to the motor onset. Moreover, the increased microglia activation in the putamen was not associated with disease severity demonstrated by neither clinical symptoms nor loss of dopaminergic terminals. Recent multimodal PET studies showed that while microglial activation was increased in brain regions such as midbrain, frontal cortex, and putamen, the regional activation levels did not correlate with the severity of motor symptoms, disease duration nor dopaminergic uptake [15, 18, 21], inconsistently, one study showed parallel changes in microglial activation and corresponding dopaminergic terminal loss [12]. It appears to suggest that the regional level of microglia activation may be an early event during neurodegeneration rather than a marker of disease progression. The initiation of microglia activation can be pre-manifesting, since evidence suggests the presence of microglia activation in the prodromal phase of PD. Stokholm et al. has reported the elevation of microglia activation in the substantial nigra of individuals with high risk for development of PD, including REM sleep behavior disorders and non-manifesting carriers with *LRRK2* G2019S mutation [30, 31]. Similarly, Mullin et al. reported increased microglia activation in the substantial nigra of carriers with *GBA* mutations [32]. Consistently, we found ^18^F-PBR06 SUVR in the brainstem was positively correlated with local ^18^F-FP-DTBZ SUVR (Table 2). However, whether the microglia activation is neuroprotective or detrimental can not be answered due to the inability to distinguish the proinflammatory M1 phenotype of microglia and the anti-inflammatory M2 microglia with TSPO-PET, as discussed by Stokholm et al. and Mullin et al. [30, 32].

The proinflammatory phenotypes, such as Th1 and Th17 cells, promote inflammation by cytokine production, while Th2 and T regulatory cells help B lymphocytes can alleviate inflammation [33]. A number of studies have demonstrated a decreased number of CD3+ and CD4+ Th lymphocytes and increased serum levels of IL-6, TNF-α, IL-1β, IL-2, and IL-10 in PD [4, 5]. Consistent with our results, a previous comprehensive study on the phenotypic and functional profile of CD4+ T cell subsets in peripheral blood of early PD found a reduced circulating Th2, Th17, Th1/17, and Treg leading to a relative increase of Th1 cells, a complex Th1-biased adaptive immune response [34]. This alteration independent from PD progression and severity suggests a systematic proinflammatory status and the contribution of Th1-related mechanisms to neuroinflammation and neurodegeneration in PD. We also found an elevation of frequencies of Th17 cells and serum level of proinflammatory IL17A and significant increase in serum level of anti-inflammatory IL-10, in agreement with the findings of increased serum levels of IL-6, TNF-α, IL-1β, IL-2, and IL-10 in PD patients from a meta-analysis of 25 studies and 2654 participants [5]. Our results, also confirmed the involvement of IL17A cytokine in the pathogenesis of PD which was reported in MPTP-injected mice model and midbrain neuron model derived from induced pluripotent stem cells [8, 9]. However, while the increase of both central microglia activation and peripheral inflammation markers was noted, no significant correlation was found between central and peripheral inflammation markers in the study. A trend of positive association was noted between the level of proinflammatory markers (IFNγ, TNFα, IL6, IL8 and IL17A) and putaminal microglia activation (Additional file 1: Fig. S1), but none of the association could survive the corrections for multiple comparisons. The lack of association was inconsistent with previous studies [35–37], possibly due to the limited number of PD participants enrolled and global normalization.

There are several limitations of the current study. Most TSPO tracers are well described with a two-tissue compartmental model using an arterial input function [38]. However, acquisition of blood samples required for a plasma input function modeling approach which could not be done in our center, thus, a clinically feasible PET quantitation measure SUVR with the average global brain SUV as a reference was adopted [29]. This method was supported by evidence that the distribution volume of ^18^F-PBR06 remained stable during 60–120 min [39], and the test–retest reliability of the SUVR approach was high [28, 29, 39, 40]. Furthermore, the global SUV was generally used to compared between patient and control which was not significant different in our study. We agree that caution should be exercised when interpreting the meaning of the TSPO SUVR in a relative regional activation of microglia compared to the entire brain. However, PD is a disease with relatively focal degeneration on the nigra-striatum regions. Another limitation was that the VOI of substantia nigra was segmented automatically with an atlas build in the MNI space. The procedure was challenging due to the small size of the anatomical structure of substantia nigra, which could introduce potential noise to the SUVR. However, the normalization of PET/MR data was closely monitored to ensure the location of substantia nigra of each individual in the MNI space, and the bias from researchers could be reduced with an atlas-based automatic VOIs comparing to VOIs drawn manually. A third limitation was that while TSPO-PET imaging is widely applied as a non-invasive marker of neuroinflammation in vivo, there are questions about specificity of elevated TSPO to activated microglia. The protein is known to be expressed by multiple cell types such as astrocytes [41]. Future development of tracers specifically targeting microglia activation is required to enable pivotal studies elucidating the role of neuroinflammation in disease onset and progression.

## Conclusions

Our results suggest microglial activation in the putamen and peripheral phenotypic Th1 bias is a characteristic of early PD. Adaptive immunity might be involved in microglia activation in the process of neurodegeneration in PD, which may be a potential biomarker for the early detection and the target for immunomodulating therapy.

## Supplementary Information

**Additional file 1: Table S1.** The ^18^F-PBR06 SUVR between PD and healthy controls. **Table S2.** Microglia activation in the subregions of putamen in PD and control. **Figure S1.** Associations between peripheral inflammation and brain microglia activation.

## Data Availability

The data that support the findings of this study are available on request from the corresponding author. The data are not publicly available due to privacy or ethical restrictions.

## Abbreviations

GDS: Geriatric Depression Scale
MDS-UPDRS III: MDS-Unified Parkinson’s Disease Rating Scale motor scores
MMSE: Mini-Mental State Examination
MoCA: Montreal Cognitive Assessment
PD: Parkinson’s disease
RBDQ-HK: REM Sleep Behavior Disorder Questionnaire-Hong Kong
SUVR: Standardized uptake value ratio
Th: T helper
TSPO: 18-kDa translocator protein
