# Supplementary material for: Brain microglia activation and peripheral adaptive immunity in Parkinson’s disease: a multimodal PET study
Source: J Neuroinflammation. 2022 Aug 29;19:209. doi: 10.1186/s12974-022-02574-z (PMC9422161; doi:10.1186/s12974-022-02574-z)

**Table S1 The ^18^F-PBR06 SUVR between PD and healthy controls**

| VOI | Control | Side | PD | P | P_ancov_ |
| --- | --- | --- | --- | --- | --- |
| Caudate | 0.90±0.06 | Ipsilateral | 0.93±0.07 | 0.166 | 0.205 |
| Putamen | 1.00±0.05 |  | 1.07±0.05 | 0.001* | < 0.001* |
| Substantia nigra | 0.88±0.10 |  | 0.91±0.09 | 0.278 | 0.073 |
| Caudate |  | Contralateral | 0.92±0.05 | 0.343 | 0.283 |
| Putamen |  |  | 1.04±0.06 | 0.065 | 0.057 |
| Substantia nigra |  |  | 0.92±0.11 | 0.215 | 0.177 |
| Brainstem | 1.02±0.06 |  | 1.01±0.07 | 0.632 | 0.911 |

* P < 0.05. P_ancov_: adjusted for age and gender. SUVR: standardized uptake value ratio.

**Table S2 Microglia activation in the subregions of putamen in PD and control**

| **VOI** | **Control** | **Side** | **PD** | **P** | **P_ancov_** |
| --- | --- | --- | --- | --- | --- |
| **Anterior dorsal putamen** | 1.00±0.05 | Ipsilateral | 1.05±0.06 | 0.004^*^ | 0.005^*^ |
| **Anterior ventral putamen** | 1.03±0.06 |  | 1.10±0.07 | 0.001^*^ | 0.001^*^ |
| **Posterior dorsal putamen** | 1.01±0.05 |  | 1.05±0.06 | 0.035^*^ | 0.062 |
| **Posterior ventral putamen** | 1.00±0.06 |  | 1.07±0.07 | 0.001^*^ | 0.001^*^ |
| **Anterior dorsal putamen** |  | Contralateral | 1.02±0.07 | 0.265 | 0.298 |
| **Anterior ventral putamen** |  |  | 1.05±0.07 | 0.224 | 0.237 |
| **Posterior dorsal putamen** |  |  | 1.04±0.09 | 0.241 | 0.357 |
| **Posterior ventral putamen** |  |  | 1.05±0.08 | 0.017^*^ | 0.015^*^ |

* P < 0.05. P_ancov_: adjusted for age and gender.

**Figure S1 Associations between peripheral inflammation and brain microglia activation**

Correlations within the PD group between ^18^F-PRB06 SUVR, cytokines and percentages of T lymphocytes. I = ipsilateral; C = contralateral; SN = substantial nigra; Th = T helper cells.


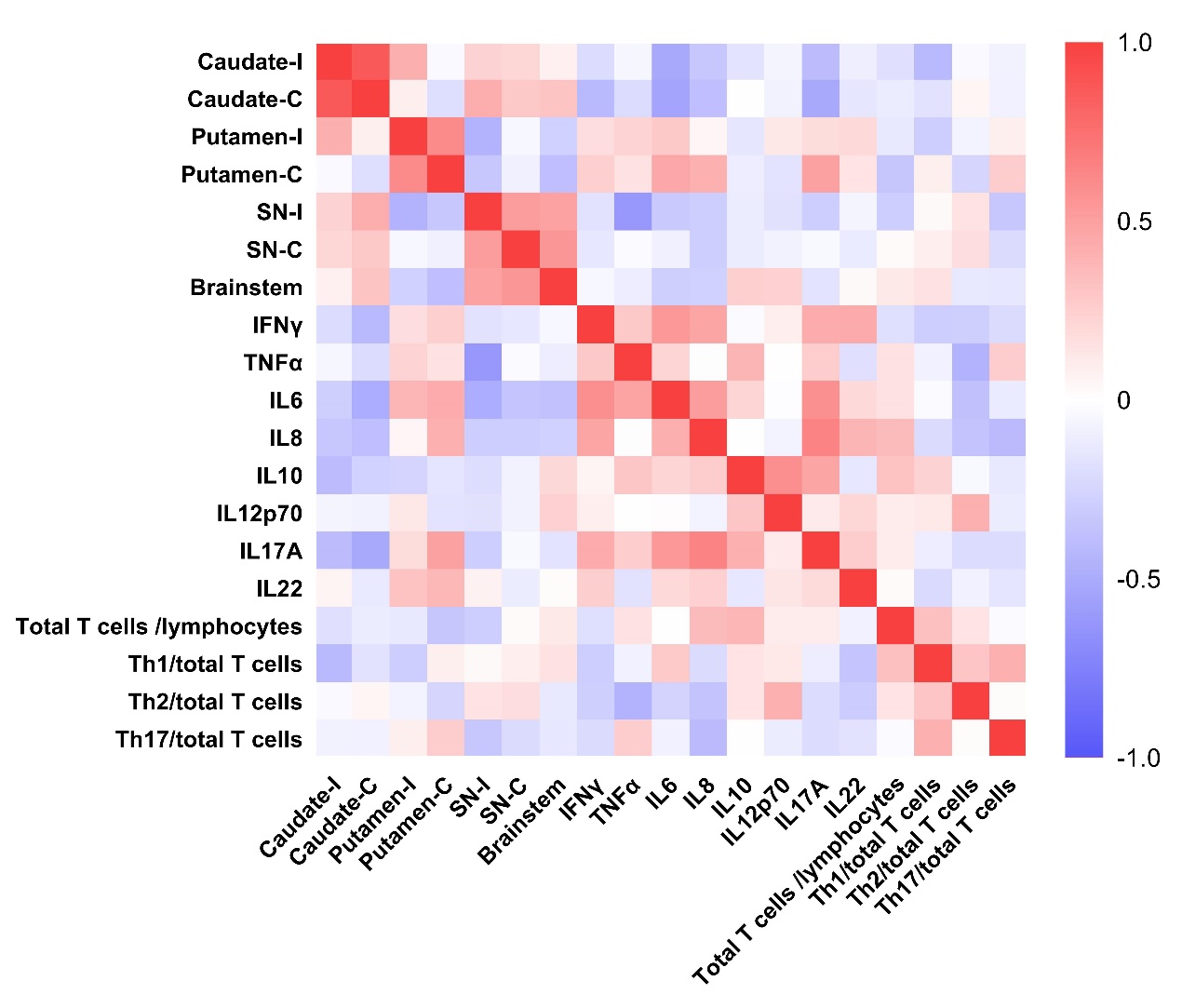

Supplement: Supplementary file 1 — Additional file 1: Table S1. The 18F-PBR06 SUVR between PD and healthy controls. Table S2. Microglia activation in the subregions of putamen in PD and control. Figure S1. Associations between peripheral inflammation and brain microglia activation. [file 12974_2022_2574_MOESM1_ESM.docx]
